# A flexible and biocompatible triboelectric nanogenerator with tunable internal resistance for powering wearable devices

**DOI:** 10.1038/srep22233

**Published:** 2016-02-26

**Authors:** Yanbo Zhu, Bin Yang, Jingquan Liu, Xingzhao Wang, Luxian Wang, Xiang Chen, Chunsheng Yang

**Affiliations:** 1 National Key Laboratory of Science and Technology on Micro/Nano Fabrication, Department of Micro/Nano Electronics, Shanghai Jiao Tong University, Shanghai, 200240, China

## Abstract

Recently, triboelectric energy nanogenerators (TENGs) have been paid the most attention by many researchers to convert mechanical energy into electrical energy. TENGs usually have a simple structure and a high output voltage. However, their high internal resistance results in low output power. In this work, we propose a flexible triboelectric energy nanogenerator with the double-side tribological layers of polydimethlysiloxane (PDMS) and PDMS/multiwall carbon nanotube (MWCNT). MWCNTs with different concentrations have been doped into PDMS to tune the internal resistance of triboelectric nanogenerator and optimize its output power. The dimension of the fabricated prototype is ~3.6 cm^3^. Three-axial force sensor is used to monitor the applied vertical forces on the device under vertical contact-separation working mode. The Prototype with 10 wt% MWCNT (Prototype I) produces higher output voltage than one with 2 wt% MWCNT (Prototype II) due to its higher dielectric parameter measured by LRC impedance analyzer. The triboelectric output voltages of Prototype I and Prototype II are 30 V and 25 V under the vertical force of 3.0 N, respectively. Their maximum triboelectric output powers are ~130 μW at 6 MΩ and ~120 μW at 8.6 MΩ under vertical forces, respectively.

In recent years, energy harvesting has been investigated not only to cope with the global energy crises, but also to realize potential energy supply for micro-actuators, micro-sensors, especially wearable and implantable medical devices. A variety of harvesters have been demonstrated to convert ambient energy into the electrical energy employed by different mechanisms, including electromagnetic^1^,^2^,^3^,^4^,^5^, electrostatic^6^,^7^,^8^,^9^,^10^, piezoelectric^11^,^12^,^13^,^14^,^15^ mechanisms. However, these harvesters based on traditional working mechanisms cannot satisfy the power supply of electronic devices. Firstly, the output powers of these devices fabricated with complex fabrication processes are quite low for their applications. Secondly, these devices are not strong enough to endure long-time exposure to the vibration motion because most of them are not fabricated with organic materials. Finally, they lack the flexibility and biocompatibility, which especially limits their application for implantable self-powered electronics. Triboelectric energy nanogenerators (TENGs)^16^,^17^,^18^,^19^,^20^,^21^,^22^,^23^,^24^,^25^ by working on coupled triboelectrification and electrostatic induction have been popularly explored very recently due to relatively high output performance and easy fabrication process. The output voltages of TENGs can reach very high through optimized structure, proper materials selection in triboelectric series^26^,^27^ and nanoscale surface modification to enhance contact area. The applications of the TENGs have been explored for self-powered sensors and wearable devices^28^,^29^,^30^,^31^. Recently, a polyvinylidene fluoride (PVDF) based triboelectric nanogenerator is proposed for wearable application^32^. TENGs based on PET and Kapton flexible organic materials with double metal electrodes have been proposed to drive small electronics^33^. Moreover, Kapton, PET and PVC (polyvinylchloride) at different positions in the triboelectric series are selected to compare their output performance. As a result, the combination of Kapton-PET or PVC-PET can achieve high-output power because Kapton and PVC are far away from the location of PET. A broadband triboelectric energy harvester with PDMS and SU-8 film patterned by micropillar and metal as friction material has been proposed with common beam structures to achieve broadband energy harvesting mechanism^34^,^35^, and the relationship of micro surface structure’s size and the voltage output is carefully studied. However, due to the very high impedance of TENGs, the low short-circuit current of the devices limits their applications. To enhance the output performance of TENGs, two or more energy conversion mechanisms nanogenerators are proposed as an effective way to compensate the disadvantage of TENGs^36^,^37^,^38^.

Here in this paper, we have developed a flexible and biocompatible triboelectric nanogenerator with simple fabrication process to convert mechanical energy into electricity, which can realize the tunable internal resistance. The tribological layers of this device are PDMS and PDMS/MWCNT. The multi-wall carbon nanotubes (MWCNTs) are doped into PDMS as one conductive triboelectric layer because PDMS has good biocompatibility^39^,^40^,^41^,^42^. MWCNT has good electrical conductivity and is deployed to tune the internal resistance by the doping concentration for matching the loading resistance. The biocompatible and electrode bonded PDMS thick film works as the other triboelectric layer. This proposed energy harvester has good flexibility, biocompatibility for the application of wearable devices.

## Results

### Structure and working principle

**Figure 1:**
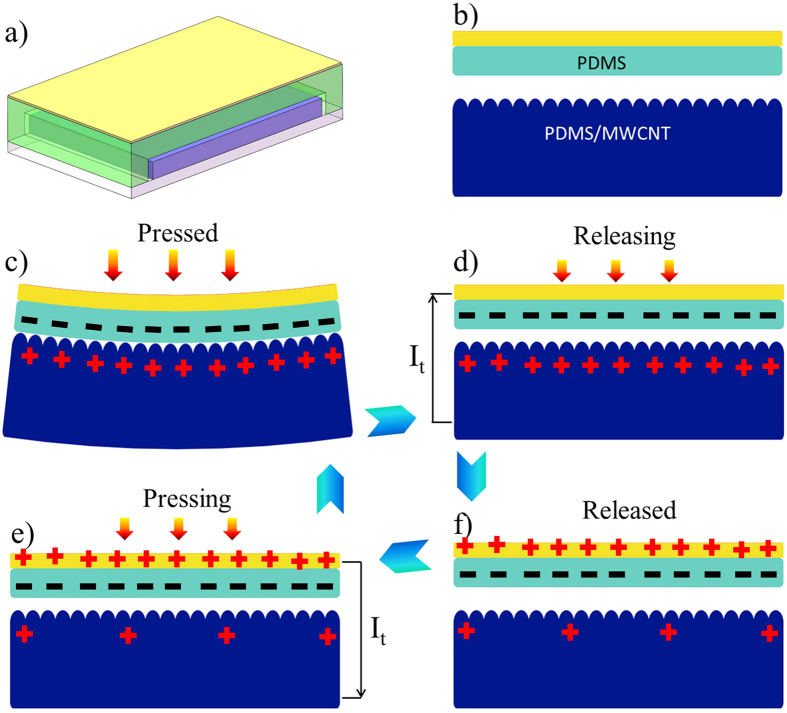
Design and working principle of the nanogenerator. (**a**) Schematic view of the nanogenerator prototype. (**b**) Three layers of the nanogenerator prototype. (**c**) Electrons are transferred from PDMS/MWCNT to PDMS when they contact with each other. (**d**) Electrons transfer between electrodes at separating state. (**e**) Vertical forces are applied on the generator again. (**f**) PDMS and PDMS/MWCNT at full-separated state.

Figure 1(a,b) show the 3-D schematic view and cross-sectional diagram of the designed nanogenerator, respectively. The generator includes two layers of PDMS with metal electrode and the composite layer of PDMS/MWCNT. PDMS/MWCNT is patterned with micro structures to increase the contacting area and improve electrical output of TENG. The two layers of the nanogenerator are insulated with a constant small separation gap at the steady equilibrium state, as shown in Fig. 1(b). The dimension is 60 mm × 20 mm, the thickness of PDMS and PDMS/MWCNT films are 1.5 mm and 1.5 mm, respectively. The maximum arc-shaped internal gap between the two triboelectric layers is 2 mm. The total volume of the final fabricated prototype is ~3.6 cm^3^.

Figure 1(c–f) demonstrate the working principle of the double-ended beam triboelectric nanogenerator. Triboelectric output is caused by the coupling between the triboelectric effect and electrostatic induction under periodic contact and separation of the two materials surfaces. The PDMS/MWCNT surface will be positively charged while the PDMS surface is with negative charges when they are brought into contact. The double-clamped PDMS beam will bend downward until it contacts the bottom PDMS/MWCNT when a vertical force is applied, as shown in Fig. 1(c). The surface charges are transferred from PDMS/MWCNT to PDMS due to triboelectrication effect because they have different charge affinity with unit of nC/J. The larger affinity difference between two triboelectric materials contributes to the larger amount of transferred charges.

While the force is releasing, electric potential difference between the PDMS and PDMS/MWCNT will drive electrons, which results in the observed output 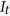 from the bottom PDMS/MWCNT layer to the top electrode, as shown in Fig. 1(d). 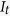 current output is related to the charges transferring between the triboelectric materials surfaces, and it can be expressed as:


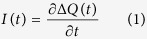


where Δ*Q* is the transferred charges.

Based on the theoretical study of the contact-mode TENGs^43^, a differential equation of the transferred charges can be expressed as:


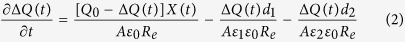


where 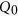 is the charges on the triboelectric material surfaces, 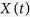 is the movement interval equation, 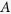 is the area of the triboelectric material surfaces, 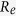 is the external load resistance, 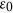 is the vacuum dielectric permittivity, 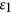 and 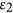 are the relative permittivity of PDMS and PDMS/MWCNT, respectively, 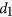 and 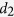 are the thickness of PDMS and PDMS/MWCNT, respectively.

**Figure 2:**
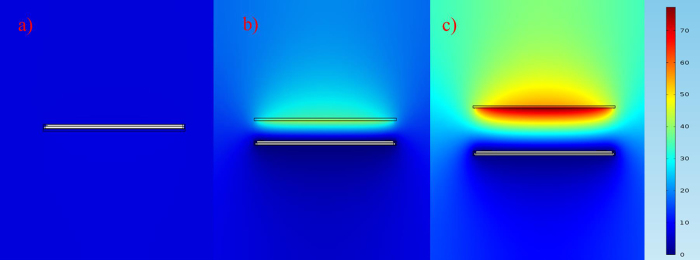
Finite-element simulation of the periodic electric potential difference between the two triboelectric materials surfaces. (**a**) Electric potential between the triboelectric material surfaces with 0.1 mm interval. (**b**) Electric potential with 5 mm interval. (**c**) Electric potential with 10 mm interval.

Furthermore, in order to reinforce the working principle, we take a further step to simulate the electric potential by virtue of COMSOL finite-element simulation software. When the nanogenerator is pressed, the two triboelectric material surfaces will be charged with the same surface density^44^. As shown in Fig. 2, the electric potential difference will also reach the maximum value when the interval between the two material surfaces arrives at the largest value. Thus the movement interval between the two triboelectric material surfaces will affect the output voltage of the nanogenerator. In Fig. 1(f), applied force is released and electrons have been accumulated on the electrode. In Fig. 1(e), electrons are driven from the top electrode to the bottom PDMS/MWCNT layer leading to a reverse triboelectric current 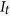 when the interval decreases.

### Material and Improvement

**Figure 3:**
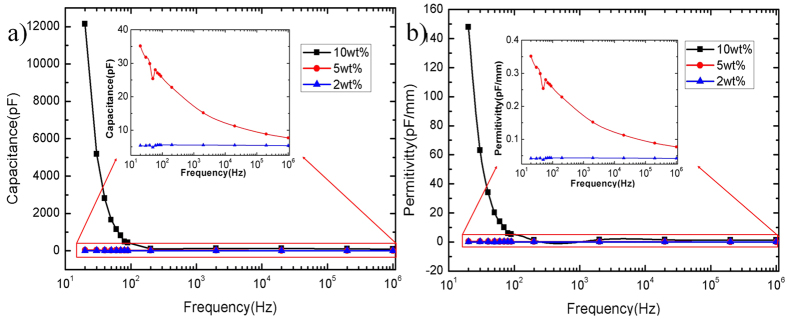
Capacitances and dielectric constants of PDMS/MWCNT. (**a**) Capacitances of fabricated 10 wt%, 5 wt% and 2 wt% PDMS/MWCNT films. Inset shows capacitances of 5 wt% and 2 wt% PDMS/MWCNT films. (**b**) Dielectric constants of fabricated 10 wt%, 5 wt% and 2 wt% PDMS/MWCNT films. Inset shows dielectric constants of 5 wt% and 2 wt% PDMS/MWCNT films.

In order to optimize the performance of the nanogenerator, different concentration the multi-wall carbon nanotubes (MWCNTs) (XFM07, Nanjing XFNANO Materials Tech Co., Ltd) are dispersed into PDMS to improve the conductivity of PDMS (Sylgard 184 A, Dow Corning Company). Due to the conductivity of PDMS/MWCNT, the triboelectric generator can work as a single-electrode TENG, which also simplify the fabrication process of the nanogenerator. Meanwhile, these films’ capacitances and dielectric constants are measured by LCR meter (WK41100, Wayne Kerr Electronics Ltd., UK), as shown in Fig. 3. It can be seen that the capacitance and the dielectric constants of 2 wt% PDMS/MWCNT are very close to ones of 5 wt% PDMS/MWCNT while the capacitance and the dielectrics constant of 10 wt% PDMS/MWCNT are much larger. Thus, 2 wt% and 10 wt% PDMS/MWCNT are selected as triboelectric layers for nanogenerators.

**Figure 4:**
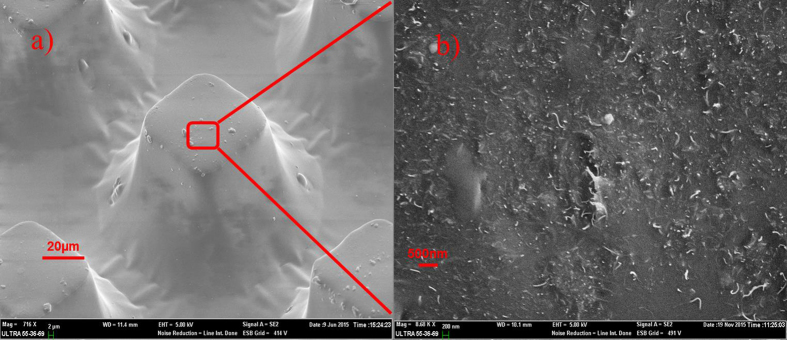
SEM photographs of MWCNTs’ dispersion in PDMS. (**a**) SEM of patterned PDMS/MWCNT films. (**b**) Enlarged SEM photograph of the MWCNTs’ dispersion in PDMS.

**Figure 5:**
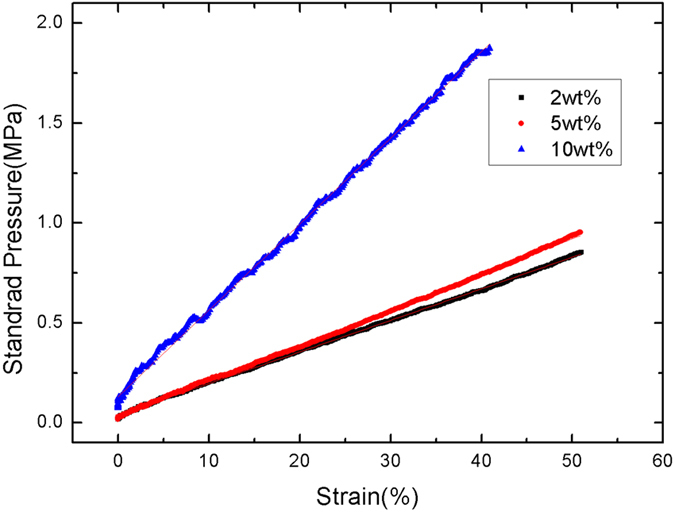
Elastic constants of 2 wt% , 5 wt% and 10 wt% PDMS/MWCNT.

**Figure 6:**
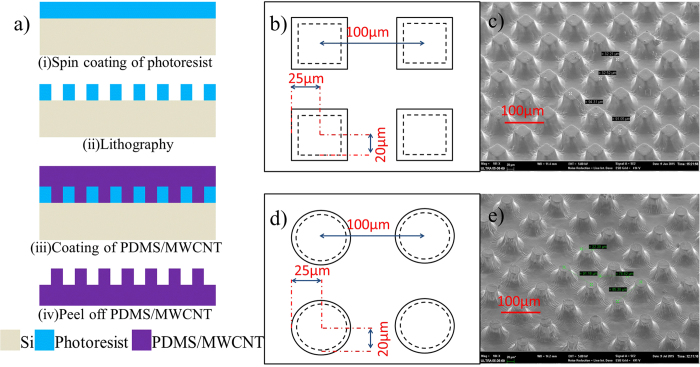
Fabrication processes and structures of micro-pillars. (**a**) Fabrication processes of micro-pillars. (**b**) Dimension of the square micro-pillars. (**c**) SEM image of the square micro-pillars. (**d**) Dimension of the circular micro-pillars. (**e**) SEM image of the circular micro-pillars.

**Figure 7:**
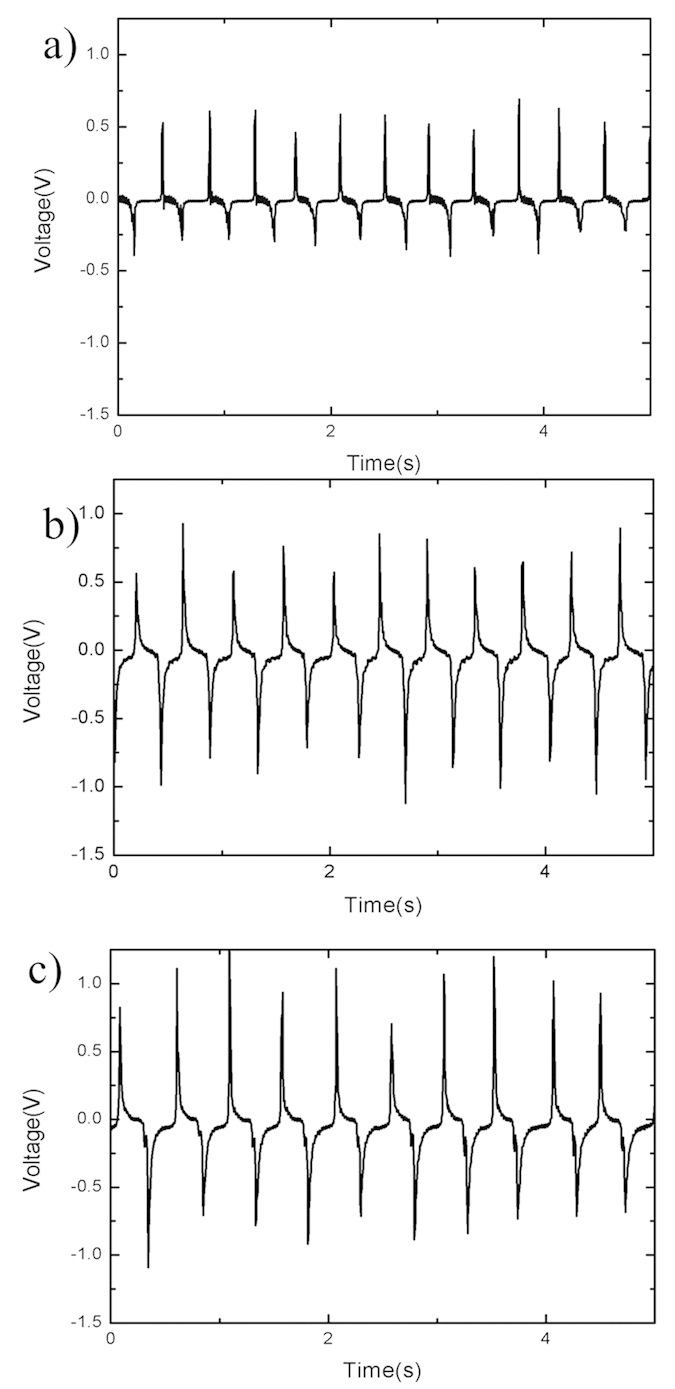
Output voltages of 2 wt% PDMS/MWCNT. (**a**) Output voltages of device without micro-pillar. (**b**) Output voltages of device with square micro-pillars. (**c**) Output voltages of device with cycle micro-pillars.

Figure 4 shows SEM images of the dispersion of MWCNTs in PDMS. It can be concluded that the MWCNTs are uniformly dispersed in PDMS. Moreover, MWCNTs as nano structures are distributed on the surface of PDMS and the surface roughness can be increased, which will contribute to improving the output performance of the nanogenerator. The elastic constant of PDMS with different doping concentration MWCNT is measured and shown in Fig. 5. It is calculated that the elastic constants of 10 wt%, 5 wt% and 2 wt% PDMS/MWCNT are 4.31 MPa, 1.78 MPa and 1.58 MPa, respectively, which shows good flexibility. Two kinds of micro structures are deployed on PDMS/MWCNT films to increase the roughness and contacting areas of PDMS/MWCNT in order to improve the output of the generators. The dimensions and SEM images of these two structures are shown in Fig. 6(b–e), respectively. In order to compare the output performance of the devices between without micro-pillars and with micro-pillars, Fig. 7 (a–c) show the output voltages with the change of times for the 2 wt% PDMS/MWCNT devices of without micropillars, square and circular micro structures under the same vertical forces, respectively. It can be seen that the device without micro structures has low output performance. It is concluded that both of the two kinds of micro-pillars increase the contacting areas of the triboelectric material surfaces and their contacting areas are almost the same, which leads to the same triboelectric output voltages.

### Output Performance

**Figure 8:**
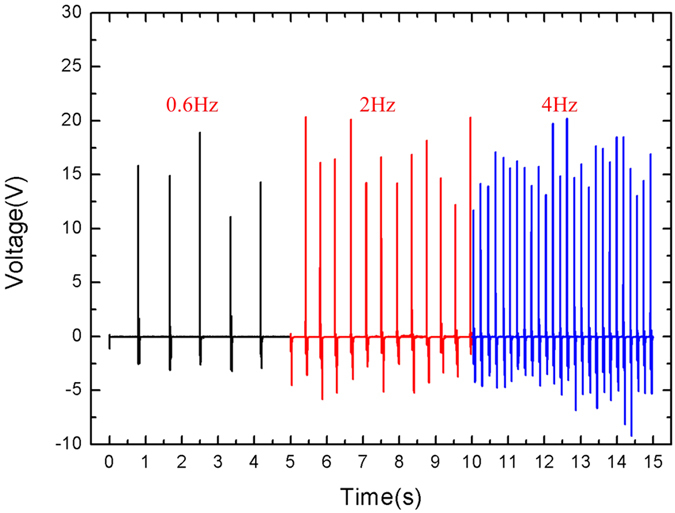
Output voltages of 2 wt% PDMS/MWCNT under different frequencies.

**Figure 9:**
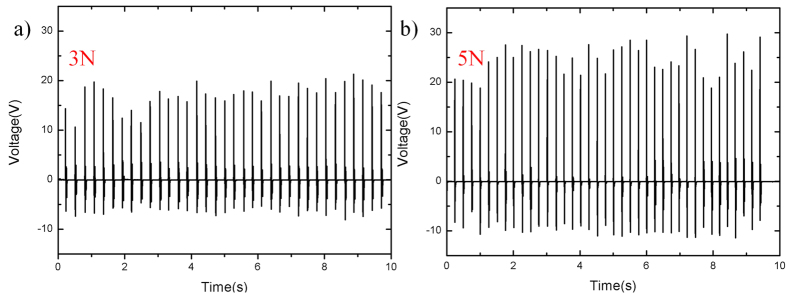
Output voltages of 2 wt% PDMS/MWCNT under different forces. (**a**) Output voltages of device under 3 N forces. (**b**) Output voltages of device under 5 N forces.

**Figure 10:**
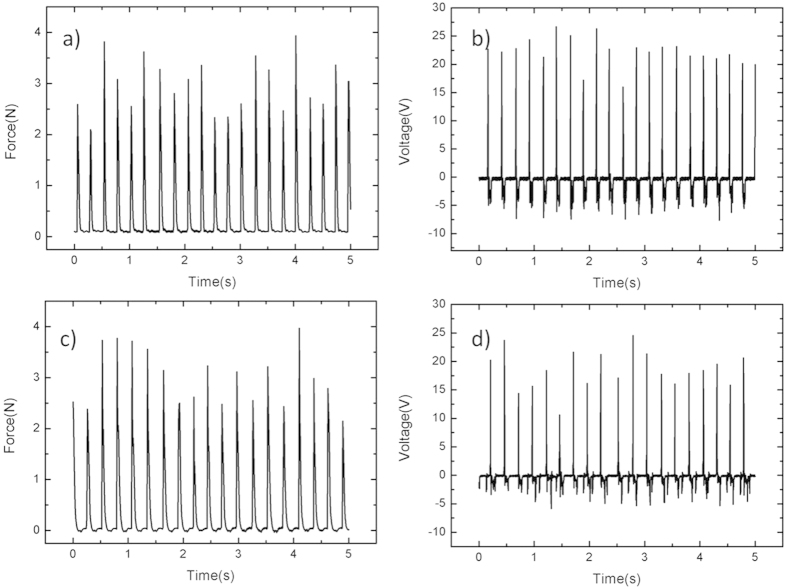
Output voltages for TENGs with 10 wt% and 2 wt% PDMS/MWCNT. (**a**) Measured vertical forces applied on the generator with 10 wt% PDMS/MWCNT (Prototype I). (**b**) Triboelectric output voltages of Prototype I. (**c**) Measured vertical forces applied on the generator with 2 wt% PDMS/MWCNT (Prototype II). (**d**) The triboelectric output voltages of Prototype II.

Figure 8 shows the output voltages of 2 wt% PDMS/MWCNT with different frequencies under the same forces. It is observed that triboelectric output voltages would increase with the increasing of the tapping force’s frequency. It may be caused by increased contact area due to the changing contacting location applied by finger under the varied frequencies. Figure 9 shows the relationship between applied forces and output voltages of the 2 wt% harvester. During experiments, the output voltages will increase with the increasing of applied forces. The reason is that large forces will cause large deformation in the tribological layers when they contact, which increases the contacting area. In addition, the large force increases the kinetic energy in the tribological layers, which causes more electrons transfer between two layers. Figure 10 shows measured forces and corresponding open-circuit output voltages from the nanogenerator of 10 wt% (Prototype I) and 2 wt% PDMS/MWCNT (Prototype II). Testing results demonstrate that the peak-peak output voltage of Prototype II is 30 V while the output of Prototype I is 25 V under the vertical force of 3 N.

**Figure 11:**
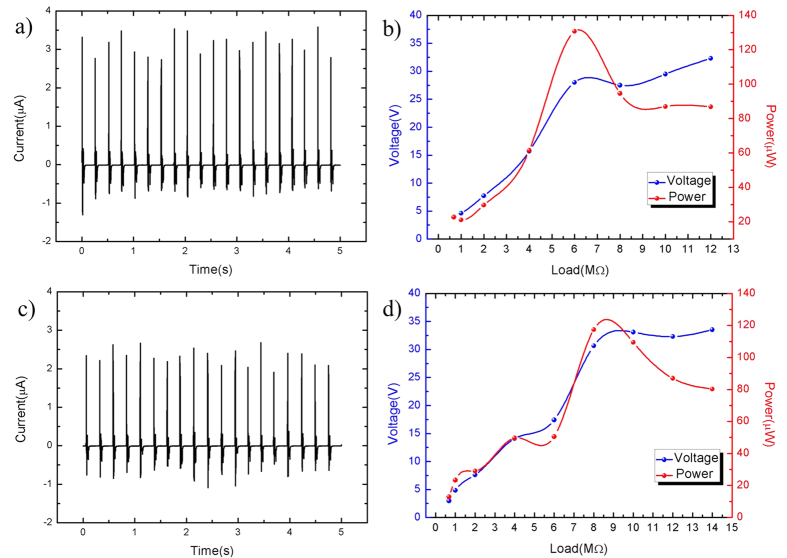
Current and power of TENGs with 10 wt% and 2 wt% PDMS/MWCNT. (**a**) Triboelectric output current of the 10 wt% nanogenerator under 6 MΩ external loads. (**b**) Triboelectric output power of the 10 wt% nanogenerator under different external loads. (**c**) Triboelectric output current of the 2 wt% nanogenerator under 8 MΩ external loads. (**d**) Triboelectric output power of the 2 wt% nanogenerator under different external loads.

To further investigate the triboelectric close-circuit output performance, the output currents with the change of times for 10 wt% and 2 wt% PDMS/MWCNT are shown in Fig. 11 (a,c), respectively. The output powers under different loading resistance are explored as shown in Fig. 11 (b,d). It can be seen that the optimized loading resistance of triboelectric of Prototype I and II are 6 MΩ and 8.6 MΩ, respectively. Their maximum triboelectric output powers are ~130 μW and ~120 μW, respectively. The power density is calculated as 0.1 W/m^2^ under small force of 3 N. The current density is about 0.3 μA/cm^2^.

The output power is calculated as following:


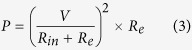


where, 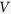 is the output voltage of the fabricated nanogenerator, 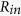 and 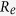 are the internal and external resistance of the device, respectively. When 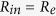, the output power of triboelectric will arrive at the maximum values. In other words, the internal resistances of Prototype I and II are 6 MΩ and 8.6 MΩ, respectively. Therefore, the internal resistance can be reduced 30%. It concludes that the 10 wt% nanogenerator has higher electric output performance than the 2 wt% nanogenerator. The contributing reason is that the 10 wt% PDMS/MWCNT film has much more MWCNT which has very good electrical conductivity at the range of low frequency. As a result, the internal resistance of the nanogenerator can be tuned by the MWCNT doping concentration.

## Discussion

**Figure 12:**
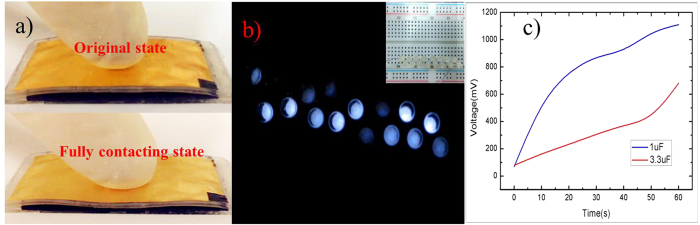
Application of TENG proposed here. (**a**) Photographs of the fabricated nanogenerator at the initial and full-contact states. (**b**) TENG is lighting 16 LED bulbs configured in parallel connection. (**c**) Charging voltage and time of the two capacitors with different capacitance values.

To validate the capacity of triboelectric nanogenerator in practical application, the nanogenerator can light LED bulbs under vertical forces from finger tap. Figure 12(a) shows the photograph of the fabricated TENG prototype under the original state and the fully contacting state, respectively. In Fig. 12(b), due to the high output performance, the generator lights up simultaneously and almost 16 light emitting diode (LED) bulbs configured in parallel connection. Figure 12(c) shows the diagram of the charging voltages and time of the two capacitors with 1 μF and 3.3 μF, respectively. By using a full-wave rectifier made by 4 diodes, the nanogenerator can power 1 μF and 3.3 μF capacitors to 1.1 V and 0.7 V in 60 seconds, respectively. The relatively low charging voltage of capacitors is related to electric energy loss in the full-wave rectifier. It is concluded that the charging of the capacitor with 1 μF capacitor is much faster than that of 3.3 μF because the 3.3 μF need more charges to reach the same voltage with the 1 μF capacitor. With the advanced development of micro-nano fabrication technologies and microelectronics circuits, some wearable devices with low-power consumption are developed to work for long time. For example, the wireless body temperature sensor system is controlled by a microcontroller unit with a power consumption of 0.18 μW in standby mode and 27 μW in active mode^45^. The CMOS image sensor for the application of retinal prostheses works with low power consumption of 0.7 μW at low frame rates^46^. This verifies that this developed nanogenerator can provide power autonomy to wearable microwatt electronic devices.

In summary, we have developed a flexible and biocompatible triboelectric nanogenerator which can work on vertical contacting mode. The output performance was carefully studied in relation to applied force, surface pattern and the MWCNT doping concentration. The operating principle was carefully demonstrated by electrostatic finite-element simulation software. The experimental results conclude that the Prototype with 10 wt% MWCNT (Prototype I) produces higher output voltage than one with 2 wt% MWCNT (Prototype II) due to its higher dielectric constants measured by LCR meter. The triboelectric output voltages of Prototype I and Prototype II are 30 V and 25 V under the vertical force of 3.0 N, respectively. Their maximum triboelectric output powers are ~130 μW at 6 MΩ and ~120 μW at 8.6 MΩ under the vertical forces, respectively. Due to the high output performance, the nanogenerators could power 16 blue LEDs once the two tribological layers contact under vertical forces. By using a full-wave rectifier made by four diodes, the nanogenerators can power 1 μF and 3.3 μF capacitors to 1.1 V and 0.7 V in 60 seconds, respectively. In summary, we have fabricated a new kind of flexible and biocompatiable TENG with tunnable internal resistance by changing the doped MWCNT concentration which has potential application for wearable devices due to its high output performance.

## Methods

### Mixture of PDMS and MWCNT

PDMS and MWCNT with the weight ratio of 10:1 and 50:1 are mixed in methylbenzene solution, respectively. Then the ultrasonic method is deployed to uniformly disperse MWCNT in PDMS until methylbenzene solution is evaporated.

### Fabrication processes of micro-pillars

Micro structures are fabricated by micro-electro-mechanical system (MEMS) process. In Fig. 6(a), firstly, a silicon substrate is prepared and 40 μm photoresist is spin-coated on it. Secondly, the square array with side length of 50 μm and circle cylinder array with diameter of 50 μm are patterned by photolithography. Finally, both the two kinds of PDMS/MWCNT coupling with curing agent in 10:1 weight ratio are coated on the pre-fabricated photoresist molds on silicon substrate, respectively. After thermo cured at 60 °C, the PDMS/MWCNT films are successfully fabricated.

### Method of output performance testing

In order to investigate the performance of TENG for harvesting mechanical energy, we use an oscilloscope (Agilent 2000X) to measure the output voltage of the two kinds of generators applied with the vertical force from finger tap which is recorded by a 3-axial force pressure sensor (ATI, NANO17). The force sensor could directly monitor the pressure value of x, y, z axes and record the force data in real time. During experiments, the bottom substrate of TENG is fixed on a plain glass pad.

## Additional Information

**How to cite this article**: Zhu, Y. *et al.* A flexible and biocompatible triboelectric nanogenerator with tunable internal resistance for powering wearable devices. *Sci. Rep.* **6**, 22233; doi: 10.1038/srep22233 (2016).

## Supplementary Material

Supplementary Video
